# Dynamic relationship between gut microbiota and post-necrotizing pancreatitis: insights from a multi-stage 16S rRNA sequencing study

**DOI:** 10.3389/fphar.2025.1577558

**Published:** 2025-05-22

**Authors:** Jiongdi Lu, Zhe Wang, Feng Cao, Jia Li, Guofeng Ji, Fei Li

**Affiliations:** ^1^ Department of General Surgery, Xuanwu Hospital, Capital Medical University, Beijing, China; ^2^ Clinical Center for Acute Pancreatitis, Capital Medical University, Beijing, China

**Keywords:** necrotizing pancreatitis, gut microbiota, 16S rRNA sequencing, inflammatory markers, microbiota-targeted interventions

## Abstract

**Background:**

Acute pancreatitis (AP) is a common digestive disorder, with necrotizing pancreatitis (NP) occurring in 20% of cases. Long-term complications can include pancreatic exocrine and endocrine insufficiency, with gut microbiota (GM) playing a significant role in pancreatic diseases. Although previous studies have established a connection between gut microbiota dysbiosis and the onset of necrotizing pancreatitis, the composition of GM in patients who have experienced post-NP post-necrotizing pancreatitis remains largely unexamined.

**Methods:**

We conducted a single-center, prospective, long-term follow-up study of 88 participants, including 68 NP patients and 20 healthy controls. NP patients were divided into NP (onset-NP) and PNP groups based on disease progression. Gut microbial diversity and composition were assessed using 16S rRNA sequencing, followed by bioinformatic analyses such as Alpha and Beta diversity metrics, linear discriminant analysis effect size (LEfSe), and functional pathway predictions. Clinical data were correlated with GM profiles to evaluate associations.

**Results:**

29.5% and 19.1% of NP patients progressed to pancreatic endocrine and exocrine insufficiency, respectively. Alpha and Beta diversity analyses revealed significantly lower microbial diversity in NP and PNP groups. Dysbiosis was characterized by a reduction in beneficial bacteria such as *Faecalibacterium prausnitzii* and *Bacteroidaceae*, and an increase in opportunistic pathogens such as *Streptococcus* and *Enterobacter*. Functional prediction suggested disruptions in cellular processes, including apoptosis and necroptosis, and links to pathways associated with inflammatory and metabolic diseases. Correlation analyses demonstrated significant associations between GM alterations and clinical markers of inflammation, such as IL-6, C-reactive protein (CRP), and Procalcitonin (PCT).

**Conclusion:**

Our findings highlight distinct GM profiles in NP and PNP patients compared to healthy controls, with partial recovery of beneficial flora in PNP patients. The study underscores the role of GM dysbiosis in NP progression and long-term outcomes, offering insights into potential therapeutic targets and strategies to improve patient management and quality of life. Future studies should explore multicenter validations and the mechanisms linking GM alterations to clinical outcomes.

## Introduction

Acute pancreatitis (AP) is a common digestive disease with an annual global incidence of 110–140 cases per 100,000 individuals (Mederos et al., 2021). Approximately 20% of AP patients progress to necrotizing pancreatitis (NP). Advancements in medical technology and clinical care have significantly reduced the mortality rate of NP (Trikudanathan et al., 2019; Leppäniemi et al., 2019; Wolbrink et al., 2020). Long-term follow-up studies of NP patients revealed that approximately 20% of post-necrotizing pancreatitis (PNP) cases develop pancreatic exocrine insufficiency (PEI) due to acinar cell injury (Lu et al., 2022). The gut microbiota (GM), a diverse community of microorganisms residing in the human intestine, consists of approximately 1,500 species and plays critical roles in human physiological metabolism and immune homeostasis regulation (Moszak et al., 2020). Clinical studies have confirmed that pancreatic exocrine function is a significant host factor influencing GM composition (Frost et al., 2019). Moreover, several studies highlight the strong association between GM and pancreatic diseases, including acute pancreatitis, chronic pancreatitis, and pancreatic cancer (Akshintala et al., 2019; Ammer-Herrmenau et al., 2020; Thomas and Jobin, 2020). GM dysbiosis and bacterial translocation in AP patients have been linked to increased risks of infectious complications, such as infected pancreatic necrosis (IPN) and bacteremia (Ammori et al., 1999; Petrov et al., 2010).

However, most existing research focuses on GM composition changes in NP patients at isolated time points or varying disease severities. Little is known about the GM composition and diversity in PNP patients. To address this gap, we investigated differences in GM composition and diversity between patients with onset NP and those with PNP. Our study aims to identify specific microbial taxa with therapeutic and predictive potential, providing a foundation for developing long-term treatment strategies for NP patients.

## Materials and methods

### Study design

This study is a prospective observational study and collected clinical data from patients with NP who admitted to Xuanwu Hospital, Capital Medical University, between 1 January 2020, and 31 July 2022. Clinical data for all patients were retrieved from the hospital’s Hitech electronic medical records system and were analyzed anonymously.

The inclusion criteria for NP patients were as follows: (1) during outpatient follow-up, patients diagnosed with PNP were included if they met the inclusion criteria after an assessment of their recovery, including physical examinations, laboratory tests, and imaging evaluations by pancreatic surgery specialists (at least associate professor level); (2) through prospective case matching, based on their previous clinical data, including ASA score (±1 point) and CT severity index (CTSI±1 point) were used to match, patients with similar disease severity to the enrolled PNP patients were selected; (3) to compare the GM composition between NP patients and healthy individuals, we included a healthy control group consisting of individuals (similar age and no underlying diseases) who underwent physical examinations at our health checkup center during the same period; (4) NP patients were subjected to regular long-term follow-up and quality-of-life assessments; (5) The NP and PNP patients included in this study all visited the General Surgery Department of Xuanwu Hospital, Capital Medical University, and belonged to the follow-up population of the same prospective and observational cohort.

### Study inclusion and exclusion criteria

The inclusion criteria for patients were as follows: (1) age ≥18 years; (2) meeting the diagnostic criteria for AP; (3) clear presence of pancreatic and/or peripancreatic necrosis on imaging (e.g., enhanced CT/MRI); (4) patients who had been discharged from the hospital and were within 3–6 months of recovery; (5) the NP patients in the acute stage (onset ≤2 weeks), and the PNP patients in the recovery stage (≥3 months after recovery).

The exclusion criteria were: (1) history of other gastrointestinal diseases (e.g., ulcerative colitis, Crohn’s disease, colorectal cancer); (2) severe neurological, cardiac, or psychiatric diseases; (3) the NP patients had received antibiotic or probiotic treatment in the first week after admission (before fecal sample collection), and the PNP patients had a history of antibiotic or probiotic use within the last month; (4) incomplete follow-up data.

### Observation outcome

The primary outcome of this study was the GM composition of NP patients at different stages (onset-NP and PNP). Secondary outcomes included: the number of patients presenting with organ failure (OF) and infected pancreatic necrosis (IPN), the number requiring surgical intervention, the type of nutritional support (enteral or parenteral), duration of nutritional support, incidence of postoperative complications (e.g., abdominal bleeding, gastrointestinal fistula, gastrointestinal obstruction), length of ICU stay, total length of hospital stay, long-term complications during follow-up (e.g., incision hernia, pancreatic pseudocyst, recurrent AP, pancreatic exocrine dysfunction, pancreatic endocrine dysfunction, chronic pancreatitis, pancreatic tumors, and other gastrointestinal symptoms), quality of life scores (Short Form-36 [SF-36], Euroqol-5 dimensions [EQ-5D] rating scales), and pain scores (Izbicki pain score). Definitions of the relevant observation indicators are provided in Supplementary Table S1.

### Patient management

Upon admission, patients received standard treatments, including trypsin inhibitors, fluid resuscitation, analgesia, and nutritional support, according to current international guidelines (Banks et al., 2013; Trikudanathan et al., 2019; Mederos et al., 2021). Antibiotics were administered only in patients with suspected or confirmed infections. Routine laboratory tests (e.g., blood counts, biochemistry, inflammatory markers) and imaging (e.g., abdominal ultrasound or CT) were conducted to monitor disease progression. If the patient’s condition improved, conservative treatment continued; if deterioration occurred, a multidisciplinary team (MDT) including pancreatic surgeons, anesthetists, intensivists, and imaging specialists provided further evaluation and treatment. Treatment adjustments were made based on the patient’s condition: (1) patients with suspected or confirmed organ failure (OF) received relevant organ support therapies (e.g., vasoactive drugs, mechanical ventilation, continuous renal replacement therapy [CRRT]); (2) patients with suspected or confirmed infections were treated with third- and fourth-generation cephalosporins or carbapenem antibiotics, adjusted based on drug sensitivity tests (Lu et al., 2019).

Indications for minimally invasive intervention included: (1) lack of improvement or worsening of the patient’s condition despite conservative treatment (e.g., increased OF, temperature, or inflammatory markers); (2) confirmed IPN; (3) worsening necrosis leading to compression of surrounding organs (e.g., digestive tract or biliary obstruction). Our pancreatic surgeons, experienced in laparoscopic necrotic tissue debridement, used minimally invasive techniques tailored to the nature, site, and integrity of the necrotic tissue, as previously described in our studies (Li et al., 2016; Cao et al., 2020; Cao et al., 2021). Following percutaneous catheter drainage (PCD) intervention, the clinical improvement (e.g., reversal of, reduction in body temperature, decreased inflammatory markers, reduced pancreatic necrosis on CT) guided the next treatment strategy.

### Follow-up

After discharge, patients were followed up via inpatient visits, outpatient visits, telephone, and email over a period of 6 months. Follow-up assessments included physical examination (e.g., incision hernia), laboratory tests (e.g., routine blood tests, biochemistry, fecal elastase-1), and imaging (e.g., enhanced CT to evaluate pancreatic morphology). Additionally, patients completed the SF-36, EQ-5D, and Izbicki pain scales to assess their quality of life. The last follow-up date for this study was 31 December 2022.

### Fecal sample collection and DNA extraction

After signing the informed consent form, fecal samples were collected within 1 week of hospitalization (routinely collected within 48 h after admission), and fresh stool samples from PNP patients meeting the inclusion criteria were retained during outpatient visits (collected on the same day). Samples were stored at −80°C within 2 h of collection. Microbial DNA was extracted using the E.Z.N.A.^®^ Soil DNA Kit (Omega Bio-tek, Norcross, GA, USA) following the manufacturer’s protocol. DNA concentration and purity were measured using a Nanodrop 2000 UV-Vis spectrophotometer (Thermo Scientific, Wilmington, USA), and DNA quality was confirmed by 1% agarose gel electrophoresis. The supplementary table shows the sampling time distribution of patients with NP and PNP.

### 16SrRNA gene sequencing and microbial analysis

Total genomic DNA was extracted using the CTAB method, and DNA concentration and purity were verified by agarose gel electrophoresis. DNA was diluted to 1 ng/μL and 16S rRNA genes (V3-V4 regions) were amplified using specific primers (341F and 806R) with barcode. PCR reactions were conducted with Phusion^®^ High-Fidelity PCR Master Mix (New England Biolabs), 2 µM of forward and reverse primers, and ∼10 ng template DNA. Thermal cycling included initial denaturation at 98°C for 1 min, followed by 30 cycles of denaturation (98°C for 10 s), annealing (50°C for 30 s), and elongation (72 °C for 30 s). The final elongation was performed at 72°C for 5 min.

Electrophoresis was conducted on a 2% agarose gel to detect PCR products. After purification with Universal DNA (TianGen, China), sequencing libraries were generated using the NEB Next^®^ Ultra DNA Library Prep Kit (Illumina, USA). Library quality was assessed on an Agilent 5400 platform (Agilent Technologies Co Ltd., United States), and sequencing was performed on an Illumina NovaSeq platform to generate 250 bp paired-end reads.

### Libraries generated and Illumina NovaSeq sequencing

Sequencing libraries were generated using NEB Next^®^ Ultra DNA Library Prep Kit (Illumina, United States) following manufacturer’s recommendations and index codes were added. The library quality was assessed on the Agilent 5400 (Agilent Technologies Co Ltd., United States). At last, the library was sequenced on an Illumina NovaSeq platform and 250 bp paired-end reads were generated.

### Statistical analysis

Previous studies in our center have found that approximately 20% of NP patients present with disorders of pancreatic exocrine and exocrine functions (Lu et al., 2022), which in turn affect the composition of the intestinal flora of the patients. Assuming α = 0.05 and β = 0.2 (efficacy 80%), the minimum sample size n required is calculated as 34 per group according to the Fless method. Statistical analyses were performed using SPSS 23.0 and GraphPad Prism 8.0. The Shapiro-Wilk test assessed normality. Normally distributed data are presented as mean ± standard deviation (M ± SD) and compared using the independent sample t-test. Skewed data are presented as median (range) and analyzed using the rank sum test. Rates were compared using the chi-square or Fisher’s exact test. Sequencing reads were grouped into operational taxonomic units (OTUs) at 97% sequence identity, and taxonomy was assigned using QIIME (version 2.0) with the Greengenes database. α-Diversity (Chao1, Shannon’s index, Simpson index) and β-diversity (PCA, PCoA) were evaluated, with Kruskal-Wallis and ANOSIM tests used for group comparisons. Linear discriminant analysis effect size (LEfSe) identified significant taxa, and paired comparisons were assessed using the Wilcoxon rank-sum test. Spearman’s rank test was used for correlation analysis. Functional prediction of the microbiome was performed using PICRUSt based on KEGG pathways, with functional differences analyzed by Kruskal-Wallis and Tukey-Kramer tests. A p-value of <0.05 was considered significant.

## Results

### Baseline characteristics and clinical outcomes

A total of 88 patients participated in this study, comprising 68 with NP and 20 healthy controls, including 50 males and 38 females, with a mean age of 48.26 ± 17.91 years. The NP patients were categorized into an onset-NP group (*n* = 34) and a post-necrotizing pancreatitis (PNP) group (*n* = 34) based on their current condition (onset or recovery phase). Detailed demographic and clinical data for the three groups are presented in Supplementary Table S2.

Among the 68 NP patients, there were 40 males and 28 females, with a mean age of 50 ± 15.42 years. The etiologies of NP included biliary pancreatitis (n = 38), hyperlipidemic pancreatitis (*n* = 21), alcoholic pancreatitis (*n* = 3), and other causes (*n* = 6, including 5 cases of post-ERCP pancreatitis and 1 case of idiopathic pancreatitis). No significant differences were observed in baseline characteristics such as gender, age, BMI, or etiology between the onset and recovery groups. Disease severity indicators, including pancreatic necrosis extent, CTSI score, and inflammatory markers at admission, were comparable between the two groups (Table 1).

**TABLE 1:** Baseline data of NP and PNP group.

Characteristics	NP group (n = 34)	PNP group (n = 34)	*P*-value
Gender [n (%)]			0.691
Male	18 (52.94)	22 (64.71)	
Female	16 (47.06)	12 (35.29)	
Age, years (M±SD)	49.94 ± 17.36	50.44 ± 13.23	0.721
BMI (kg/m^2^)	26.16 ± 5.17	25.52 ± 2.91	0.142
Pre-existing comorbidities [n (%)]			0.310
Hypertension	12 (35.29)	12 (35.29)	
Coronary heart disease	3 (8.82)	4 (11.76)	
Diabetes	7 (20.59)	7 (20.59)	
Others	12 (35.29)	15 (44.12)	
Smoking [n (%)]	7 (20.59)	6 (17.64)	
Drinking [n (%)]	9 (26.47)	10 (29.41)	
Etiology [n (%)]			0.352
Gallstones	17 (50.00)	21 (61.76)	
Hyperlipidemia	12 (35.29)	9 (26.47)	
Others	5 (14.70)	4 (11.76)	
ASA score [median (range)]	2 (1–3)	2 (1–3)	0.315
Admission temperature (M±SD)	37.21 ± 0.78	37.12 ± 0.90	0.649
CTSI score [median (range)]	8 (4–10)	8 (4–10)	0.628
Extent of necrosis [n (%)]			0.446
<30%	14 (41.76)	12 (35.29)	
30%–50%	9 (26.47)	10 (29.41)	
>50%	10 (29.41)	11 (32.35)	
Admission laboratory indicators [mean ± SD]			
WBC (×10^9^/L)	10.54 ± 4.47	9.69 ± 4.95	0.458
Percentage of neutrophils (%)	80.11 ± 12.02	75.05 ± 15.46	0.137
Hb (g/L)	114.67 ± 34.37	104.05 ± 26.35	0.158
Alb (g/L)	32.11 ± 5.90	29.99 ± 5.24	0.122
CRP (mg/L)	154.32 ± 118.46	123.40 ± 118.46	0.321
PCT (ng/mL)	1.29 ± 0.12	1.24 ± 0.42	0.592
IL-6 (pg/mL)	207.52 ± 125.69	199.62 ± 142.27	0.269

Clinical outcomes were similar between the onset and recovery groups. Persistent organ failure (POF) occurred in 13 (38.24%) and 14 (41.76%) patients, respectively (*P* = 0.587), while infected IPN was observed in 23 (67.65%) and 20 (58.82%) patients, respectively (*P* = 0.316). The average hospital stay duration was 42.62 ± 37.56 days for the NP and 54.71 ± 41.37 days for the PNP (*P* = 0.189) (Supplementary Table S3). By the last follow-up (31 December 2022), no deaths or missed follow-ups were reported. Quality of life measures, including SF-36, EQ-5D, and Lzbicki pain scores, showed no significant differences between the groups (Supplementary Table S4). Long-term complications, such as pancreatic pseudocysts, incisional hernias, recurrent acute pancreatitis, new-onset pancreatic endocrine insufficiency, exocrine insufficiency, and chronic pancreatitis, were also comparable (Table 2).

**TABLE 2:** The long-term complication between the two groups during the follow-up period.

Characteristics	NP group (n = 34)	PNP group (n = 34)	*P*-value
Follow-up time (months)	20.13 ± 10.45	21.07 ± 7.92	0.258
Long-time complications [n (%)]			
Recurrent pancreatitis	4 (11.76)	5 (14.71)	0.720
Incision hernia	2 (5.88)	1 (2.94)	0.555
Pseudocyst	4 (11.76)	4 (11.76)	0
New onset endocrine insufficiency [n (%)]			0.425
Oral medication	10 (29.41)	7 (20.59)	
Insulin	2 (5.88)	1 (2.94)	
Pancreatic exocrine insufficiency [n (%)]			0.770
Diet adjustment	1 (2.94)	1 (2.94)	
Enzyme use	6 (17.64)	5 (14.71)	
Chronic pancreatitis [n (%)]	1 (2.94)	3 (8.82)	0.614
Pancreatic cancer [n (%)]	0 (0)	0 (0)	0
Clinical symptoms [n (%)]			0.576
Bloating	3 (8.82)	5 (14.71)	
Weight loss	7 (20.59)	2 (5.88)	

### Alterations in gut microbiota in NP patients

**FIGURE 1:**
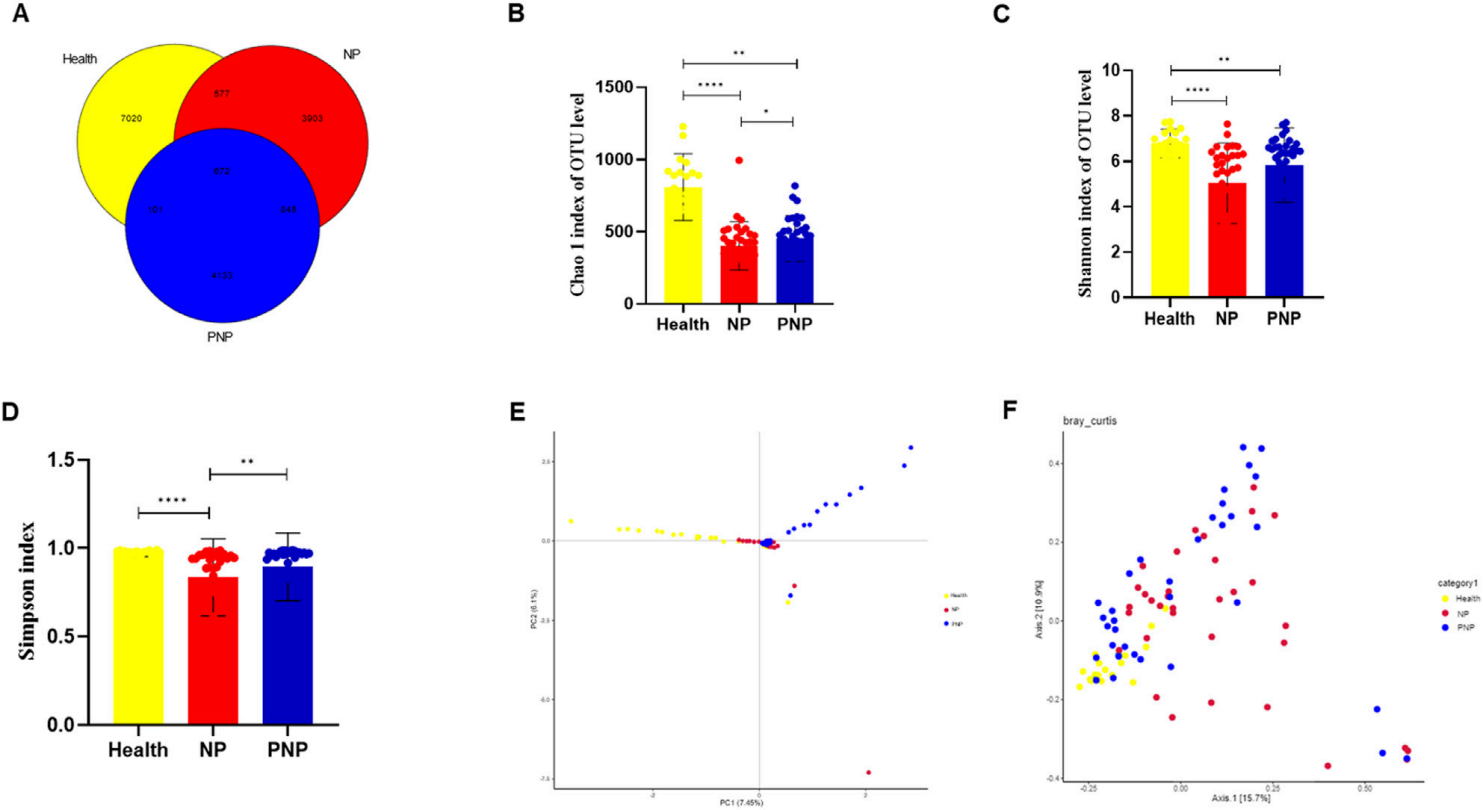
Alterations in gut microbiota in NP patients **(A)** Common and specific flora among the three groups. **(B–D)** Comparison of alpha diversity among the three groups (Chao 1 index, Shannon and Simpson indices, Wilcoxon rank sum test) **(E,F)** Comparison of beta diversity among the three groups (PCA and PCoA analysis) (*P < 0.05; **P < 0.01; ***P < 0.001).

The sequencing of all samples generated 7,379,854 base reads, from which 17,251 operational taxonomic units (OTUs) were identified, spanning 1 domain, 2 kingdoms, 42 phyla, 114 orders, 242 families, 441 genera, and 314 species. Venn diagram analysis showed 7020 OTUs unique to the healthy group, 3903 unique to the onset-NP group, 4133 unique to the PNP group, and 672 shared among all groups (Figure 1A). Alpha diversity, as measured by the Chao1 diversity index and Shannon index, was significantly lower in the onset and recovery groups compared to healthy controls (*P* < 0.05) (Figures 1B–D). Beta diversity analysis, using principal component analysis (PCA) and principal coordinates analysis (PCoA), revealed significant intergroup differences (Bray-Curtis distance algorithm; ANOSIM R2 = 0.05302, P = 0.001; R2 = 0.017512, P = 0.011) (Figures 1E,F).

### Identification of key microorganisms for differentiating in three groups

**FIGURE 2:**
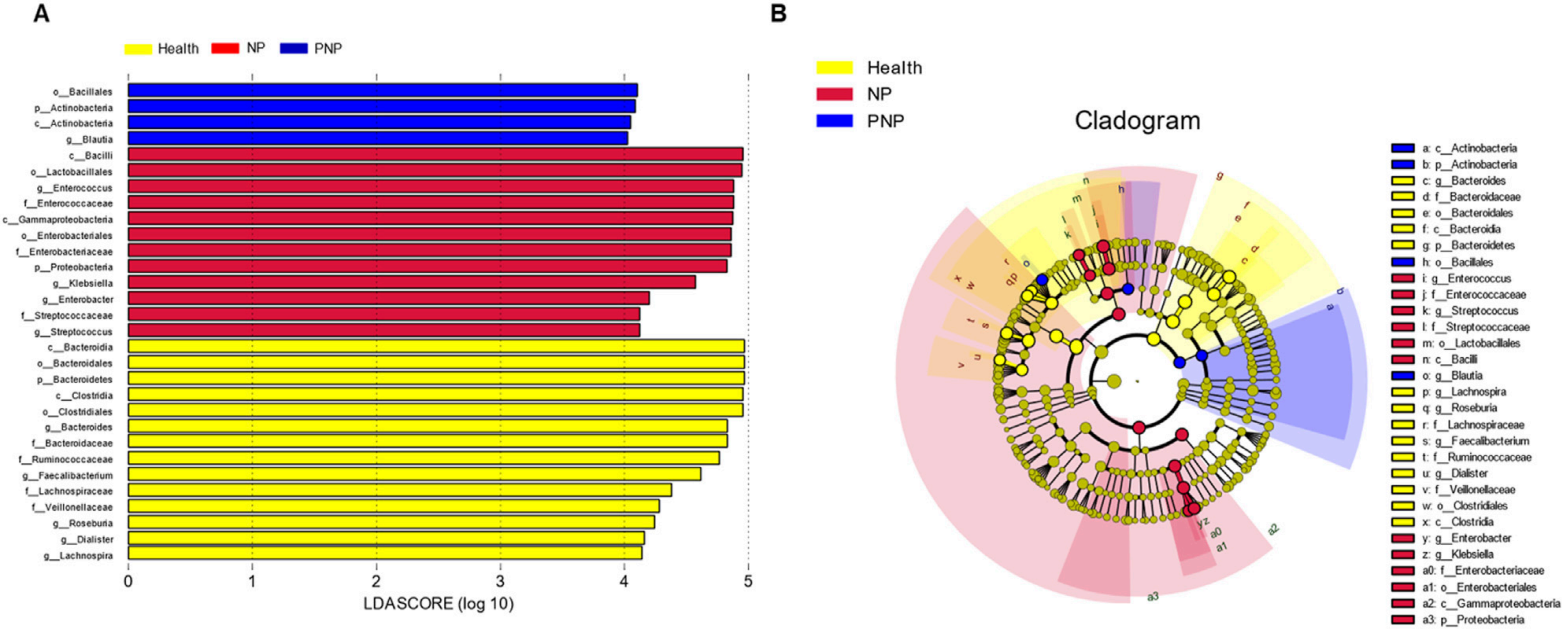
Relative abundance difference flora of patients in three groups **(A)** LDA plots of three groups of patients, each horizontal bar represents a species, and the length of the bar corresponds to the LDA value, with higher LDA values resulting in greater variation. The color of the bar corresponds to the group of microorganisms that characterize the species, and the characteristic microorganisms indicate the relatively higher abundance in the corresponding group. **(B)** Cladogram plots of the three groups of patients from the inside to the outside, corresponding to different taxonomic levels of the kingdoms, phyla, orders, and families, with the lines between levels representing affiliation. Each circle node represents a species, and a yellow node means that the difference between groups is not significant, while a non-yellow node means that the species is a characteristic microorganism of the corresponding color group (with significantly higher abundance in that group). The colored sectors mark the subordinate taxonomic intervals of the characteristic microorganisms.

LEfSe identified microorganisms with a Linear Discriminant Analysis (LDA) score >4 as characteristic of each group. The healthy group was enriched in *Lachnospira, Dialister, Roseburia, Veillonellaceae, Lachnospiraceae, Faecalibacterium, Ruminococcaceae, Bacteroidaceae, Bacteroides*, *Clostridiales*, *Clostridia*, *Bacteroidetes*, *Bacteroidales*, and *Bacteroidia*. Conversely, the morbidity group was characterized by increased *Streptococcus*, *Streptococcaceae*, *Enterobacter*, *Klebsiella*, *Proteobacteria*, *Enterobacteriaceae*, *Enterobacteriales*, *Gammaproteobacteria*, *Enterococcaceae*, *Enterococcus*, *Lactobacillales*, and *Bacilli*. The recovery group was distinguished by the presence of *Blautia, Actinobacteria, and Bacillales* (Figure 2).

### Composition of intestinal flora at different levels in the three groups

**FIGURE 3:**
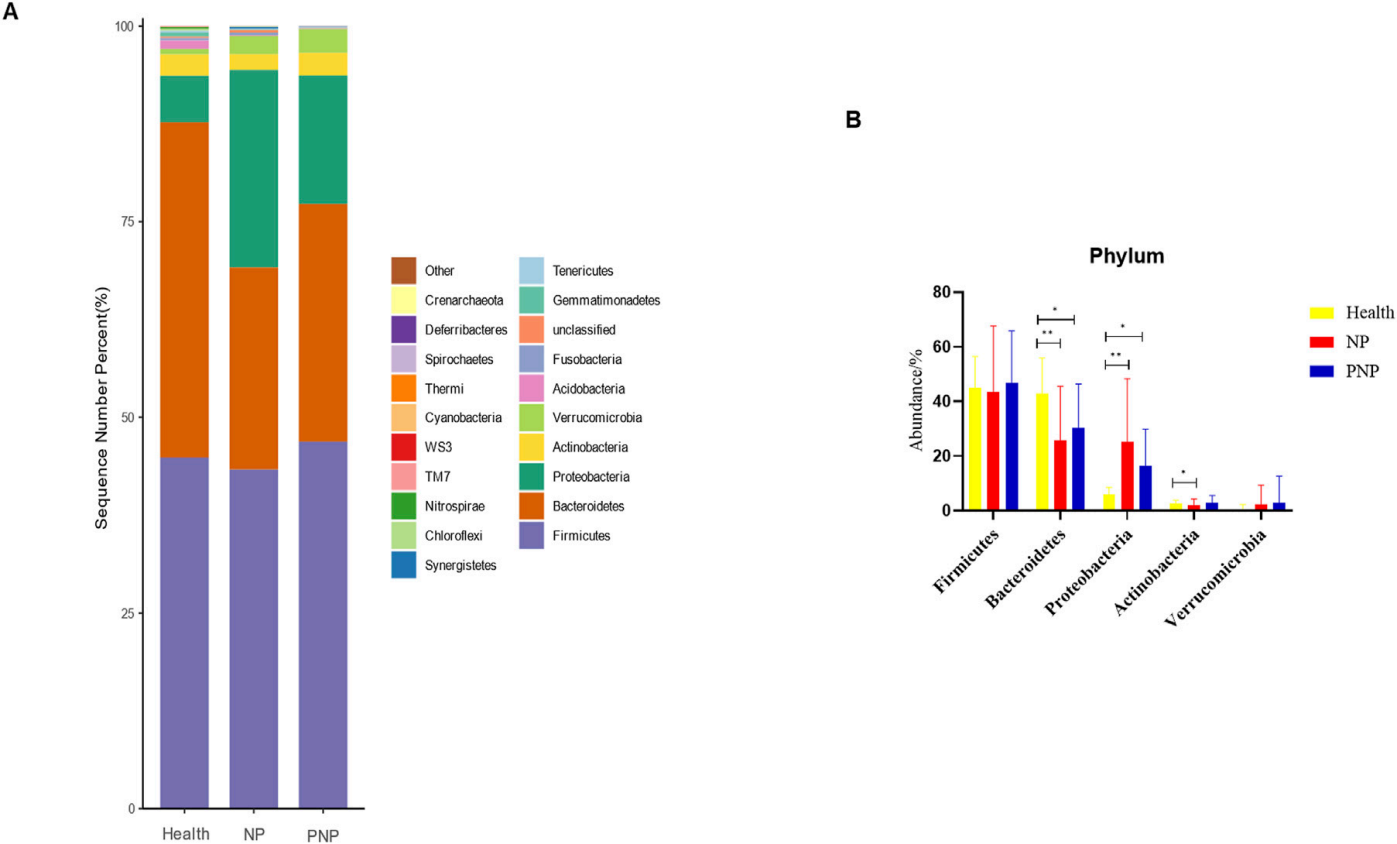
Abundance distribution of GM at the phylum level in three groups **(A)** The 20 most dominant phyla in the three groups of patients, the horizontal coordinate is the group name, the vertical coordinate (Sequence Number Percent) indicates the ratio of the number of sequences annotated to the gate level to the total annotated data, the color order of the bars corresponds to the color order of the legend on the right. Sequences without annotations are classified as unclassified, and the remaining species with low relative abundance are classified as Other. annotations from left to right and from top to bottom indicate Other (<0.05), Crenarchaeota, Deferribacteres, Spirochaetes, Thermi, Cyanobacteria, WS3, TM7, Nitrospirae, Chloroflexi, Synergistetes, Tenericutes, Gemmatimonadetes, Unclassified, Fusobacteria, Acidobacteria Verrucomicrobia, Actinobacteria, Proteobacteria, Bacteroidetes, Firmicutes; **(B)** the five most dominant phyla in the three groups (*P < 0.05; **P < 0.01; ***P < 0.001).

**FIGURE 4:**
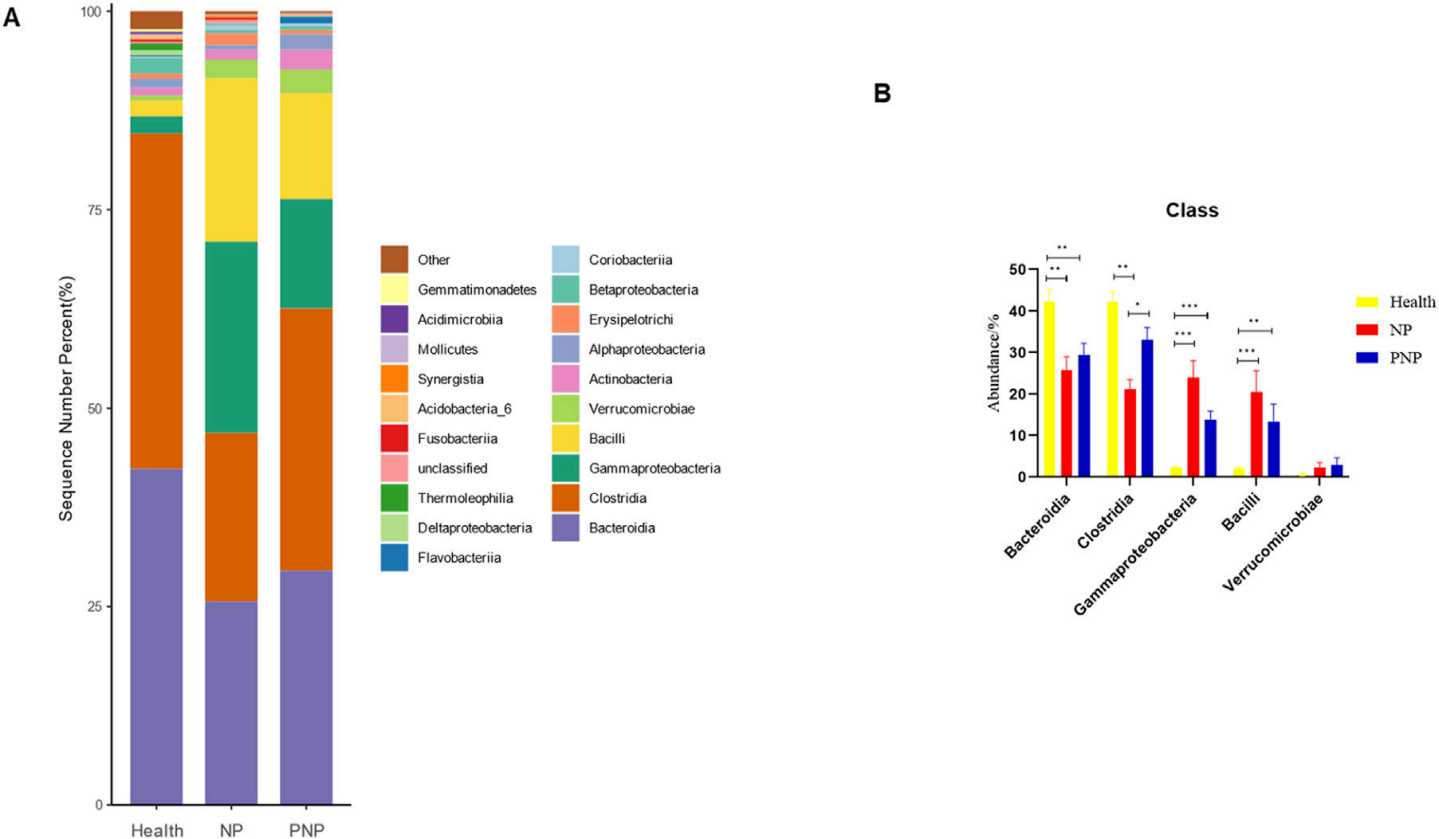
Abundance distribution of GM at the class level in three groups **(A)** The 20 most dominant compendial species in three groups of patients, the horizontal coordinate is the group name, the vertical coordinate (Sequence Number Percent) indicates the ratio of the number of sequences annotated to the compendial level to the total annotated data, and the color order of the bars corresponds to the color order of the legend on the right. Sequences not annotated at the compendial level are classified as Unclassified, and the remaining species with low relative abundance are classified as Other. annotations from left to right and from top to bottom indicate Other (<0.05), Gemmatimonadetes, Acidimicrobiia, Mollicutes Synergistia, Acidobacteria_6, Fusobacteriia, Unclassified, Thermoleophilia, Deltaproteobacteria δ, Flavobacteriia, Coriobacteriia Betaproteobacteria, Erysipelotrichi, Alphaproteobacteria, Actinobacteria, Verrucomicrobiae, Bacilli, Gammaproteobacteria, Clostridia Bacteroidia.) **(B)** The five most dominant phyla in the three groups of patients (*P < 0.05; **P < 0.01; ***P < 0.001).

At the phylum level, a total of 20 phyla were identified across the three patient groups. The most abundant phyla were *Firmicutes*, *Bacteroidetes*, *Proteobacteria*, *Actinobacteria*, and *Verrucomicrobia*. While the relative abundances of *Firmicutes*, *Actinobacteria*, and *Verrucomicrobia* were comparable among the groups, the abundance of *Bacteroidetes* decreased, and that of *Proteobacteria* increased in the NP and PNP groups compared with the healthy group, with statistically significant differences (*P* < 0.05) (Figure 3; Supplementary Table S5). At the class level, 20 classes were identified, with the most abundant being *Bacteroidia*, *Clostridia*, *Gammaproteobacteria*, *Bacilli*, and *Verrucomicrobiae*. The relative abundance of *Verrucomicrobiae* was consistent across the groups, but the NP and PNP groups showed reduced *Bacteroidia* and increased *Gammaproteobacteria* and *Bacilli* compared to the healthy group, with statistically significant differences (*P* < 0.05) (Figure 4; Supplementary Table S6).

**FIGURE 5:**
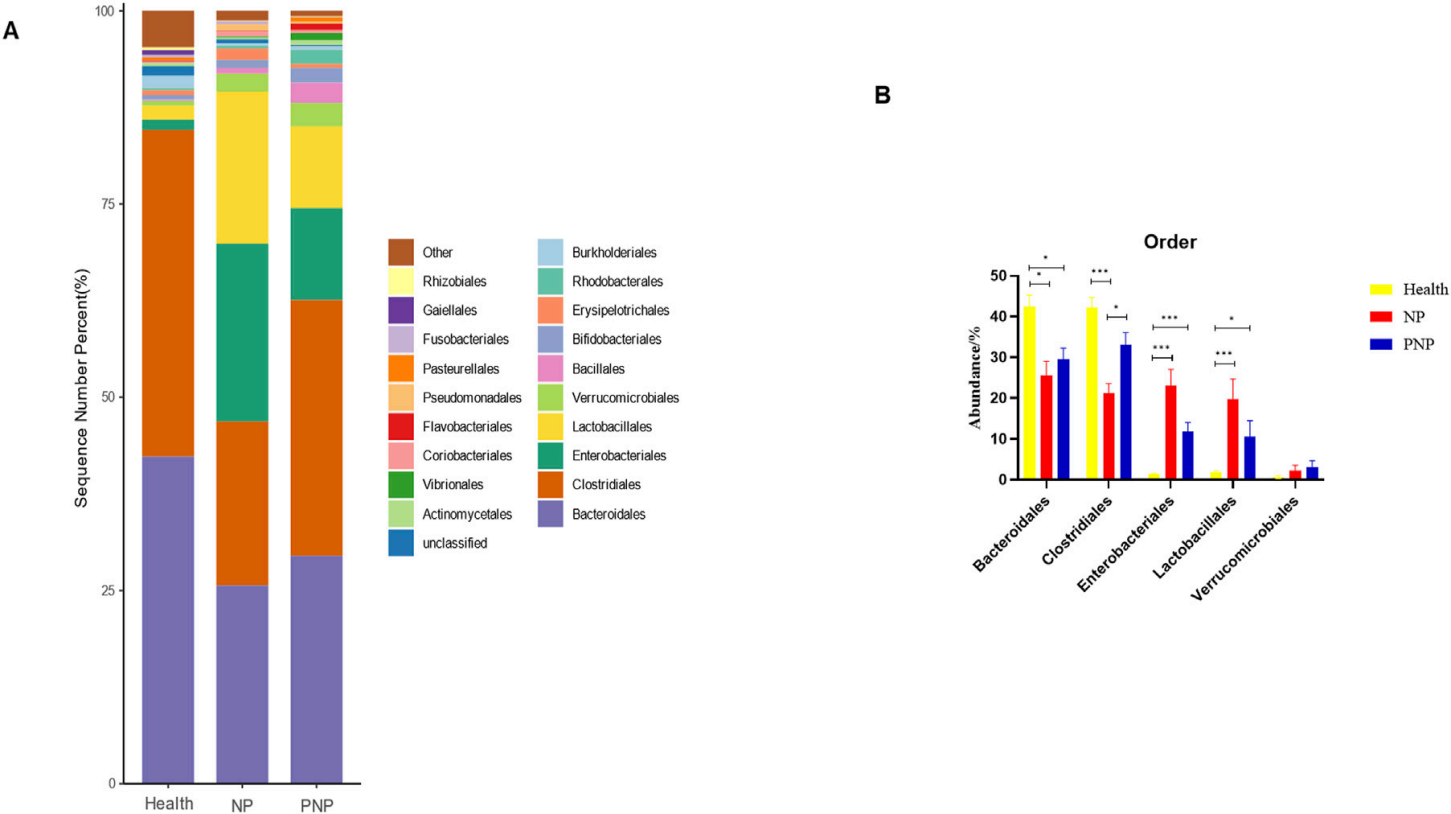
Abundance distribution of GM at the order level in the three groups **(A)** The 20 most dominant orders in the three groups of patients, the horizontal coordinate is the group name, the vertical coordinate (Sequence Number Percent) indicates the ratio of the number of sequences annotated to the order level to the total annotated data, the color order of the bars corresponds to the color order of the legend on the right. Sequences without annotations at the eye-level are classified as Unclassified, and the remaining species with low relative abundance are classified as Other. annotations from left to right and from top to bottom indicate Other (<0.05), Rhizobiales, Gaiellales, Fusobacteriales Pasteurellales, Pseudomonadales, Flavobacteriales, Coriobacteriales, Vibrionales, Actinomycetales, Unclassified, Burkholderiales Rhodobacterales, Erysipelotrichales, Bifidobacteriales, Bacillales, Verrucomicrobiales, Lactobacillales, Enterobacteriales, Clostridiales, Bacteroidales; **(B)** the five most dominant orders in the three groups (*P < 0.05; **P < 0.01; ***P < 0.001).

**FIGURE 6:**
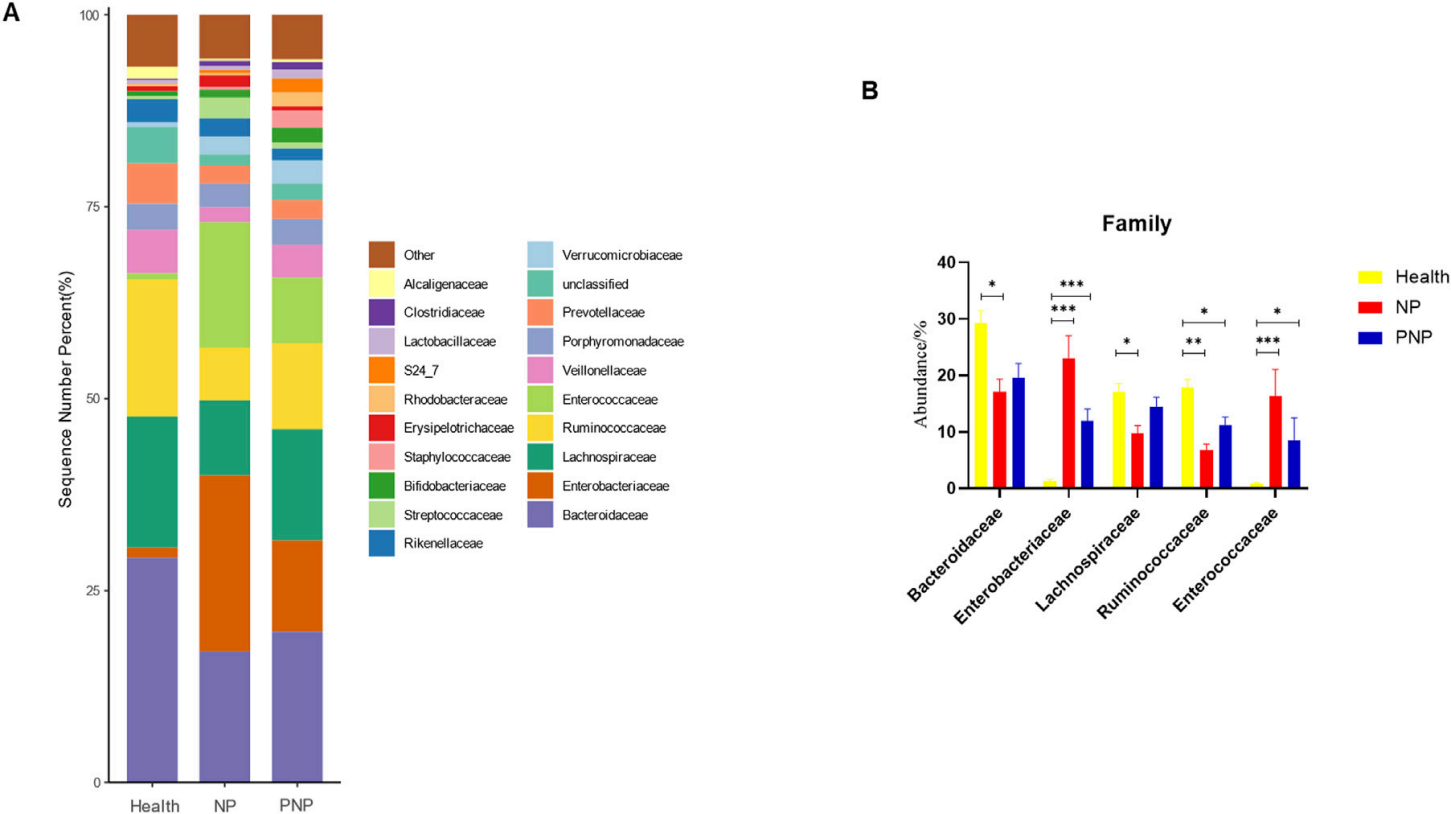
Abundance distribution of GM at the family level in the three groups **(A)** The 20 most dominant families in the three groups of patients, the horizontal coordinate is the group name, the vertical coordinate (Sequence Number Percent) indicates the ratio of the number of sequences annotated to the order level to the total annotation data, the color order of the bars corresponds to the color order of the legend on the right. Sequences without annotation at the family level are classified as Unclassified, and the remaining species with low relative abundance are classified as Other. annotations from left to right and from top to bottom indicate Other (<0.05), Alcaligenaceae, Clostridiaceae, Lactobacillaceae, S24_7, Rhodobacteraceae, Erysipelotrichaceae, Staphylococcaceae, Bifidobacteriaceae, Streptococcaceae, Rikenellaceae, Verrucomicrobiaceae, Unclassified, Prevotellaceae, Porphyromonadaceae, Veillonellaceae, Enterococcaceae, Ruminococcaceae, Lachnospiraceae, Enterobacteriaceae, Bacteroidaceae; **(B)** the five most dominant families in the three groups of patients (*P < 0.05; **P < 0.01; ***P < 0.001).

**FIGURE 7:**
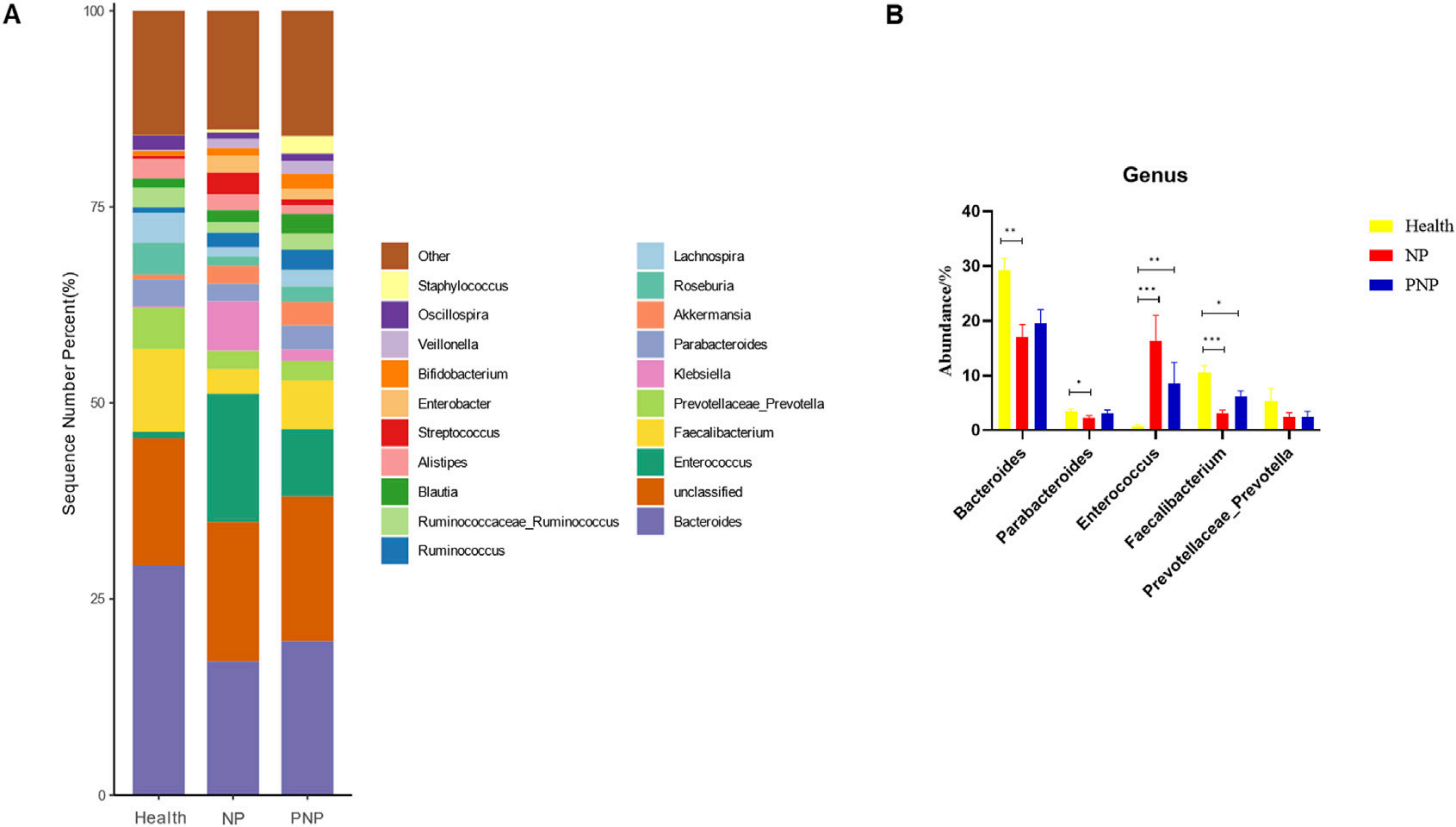
Abundance distribution of GM at the genus level in the three groups **(A)** The 20 most dominant genera in the three groups of patients, the horizontal coordinate is the group name, the vertical coordinate (Sequence Number Percent) indicates the ratio of the number of sequences annotated to the genus level to the total annotation data, and the color order of the bars corresponds to the color order of the legend on the right. Sequences not annotated at the genus level are classified as Unclassified, and the remaining species with low relative abundance are classified as Other. annotations are indicated from left to right and from top to bottom for Other (<0.05), *Staphylococcus*, Oscillospira, Veillonella Bifidobacterium, *Enterobacter*, *Streptococcus*, Alistipes, Blautia, Ruminococcaceae_Ruminococcus, Ruminococcus, Lachnospira, Roseburia Akkermansia, Parabacteroides, *Klebsiella*, Prevotellaceae_Prevotella, Faecalibacterium, *Enterococcus*, Unclassified, *Bacteroides*; **(B)** Abundance of major strains in the three groups (*P < 0.05; **P < 0.01; ***P < 0.001).

**FIGURE 8:**
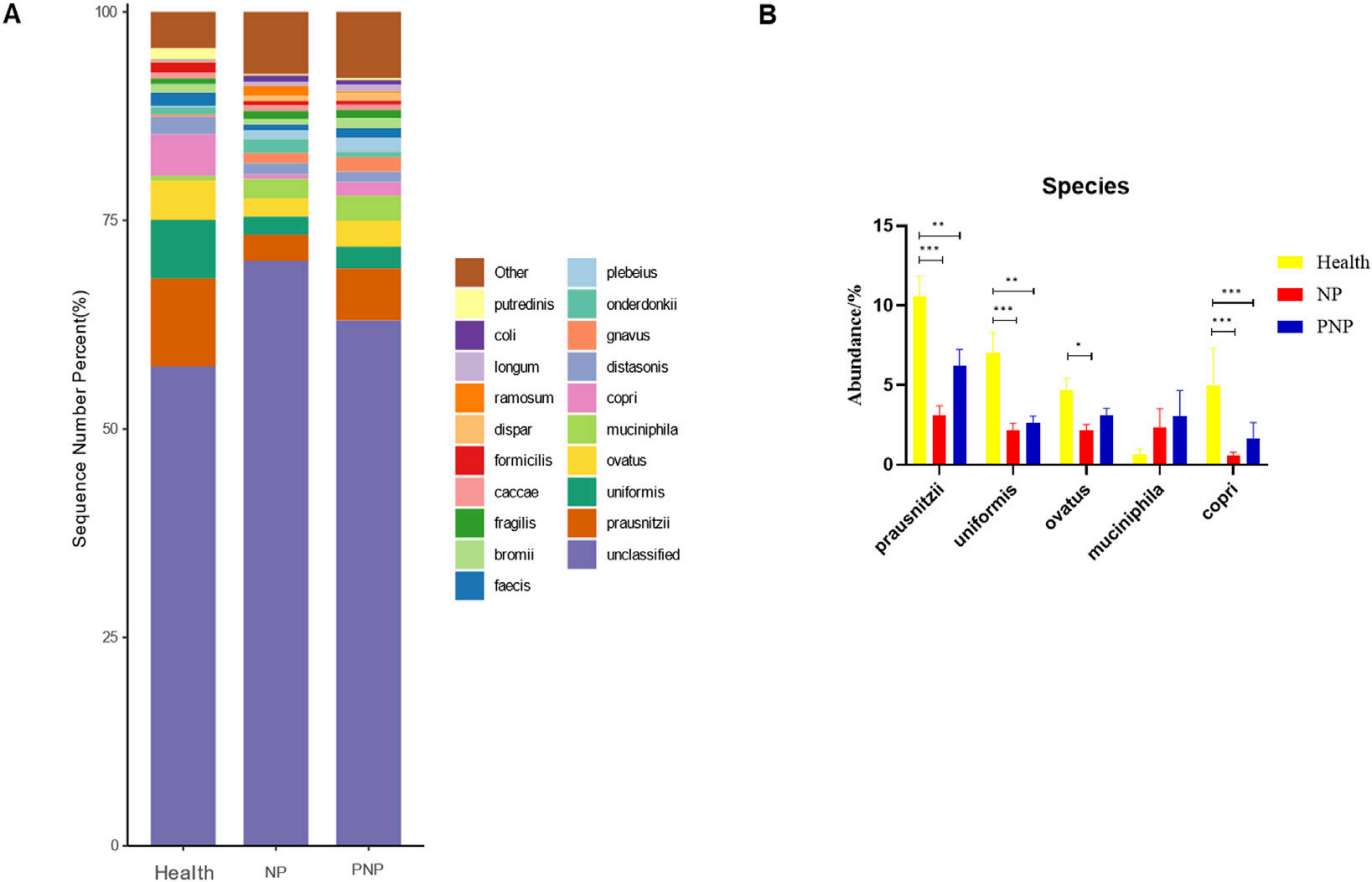
Abundance distribution of GM at the species level in three groups **(A)** The 20 most dominant species in the three groups of patients, the horizontal coordinate is the group name, the vertical coordinate (Sequence Number Percent) indicates the ratio of the number of sequences annotated to the species level to the total annotated data, the color order of the bars corresponds to the color order of the legend on the right. Sequences without annotation at the species level are classified as Unclassified, and the remaining species with low relative abundance are classified as Other. annotations from left to right and from top to bottom indicate Other (<0.05), Putredinis, coli, longum, ramosum, dispar, formicilis caccae, fragilis, bromii, faecis, plebeius, onderdonkii, gnavus, distasonis, copri, muciniphila, ovatus, uniformis, prausnitzii, unclassified **(B)** The five most dominant strains in the three groups of patients (*P < 0.05; **P < 0.01; ***P < 0.001).

At the order level, 20 orders were annotated. The most abundant orders included *Bacteroidales*, *Clostridiales*, *Enterobacteriales*, *Lactobacillales*, and *Verrucomicrobiales*. While the abundance of *Verrucomicrobiales* remained consistent, the NP and PNP groups demonstrated a decrease in *Bacteroidales* and an increase in *Enterobacteriales* and *Lactobacillales* compared with the healthy group, with statistically significant differences (*P* < 0.05) (Figure 5; Supplementary Table S7). At the family level, 20 families were identified, with the most abundant being *Enterococcaceae*, *Ruminococcaceae*, *Lachnospiraceae*, *Enterobacteriaceae*, and *Bacteroidaceae*. Compared with the healthy group, the NP and PNP groups exhibited a decrease in *Ruminococcaceae* and an increase in *Enterococcaceae* and *Enterobacteriaceae*, with statistically significant differences (*P* < 0.05) (Figure 6; Supplementary Table S8). At the genus level, 20 genera were annotated. The most abundant genera included *Klebsiella*, *Prevotellaceae_Prevotella*, *Faecalibacterium*, *Enterococcus*, and *Bacteroides*. The NP and PNP groups displayed a significant reduction in *Faecalibacterium* and an increase in *Enterococcus* compared with the healthy group (*P* < 0.05) (Figure 7; Supplementary Table S9). At the species level, 20 species were annotated, with the most common being *copri*, *muciniphila*, *ovatus*, *uniformis*, and *prausnitzii*. Compared with the healthy group, the NP and PNP groups showed a significant decrease in *prausnitzii*, *uniformis*, and *copri* (*P* < 0.05) (Figure 8; Supplementary Table S10).

### Correlation between clinical parameters and gut microbiota analysis of clinical parameters with intestinal flora

**FIGURE 9:**
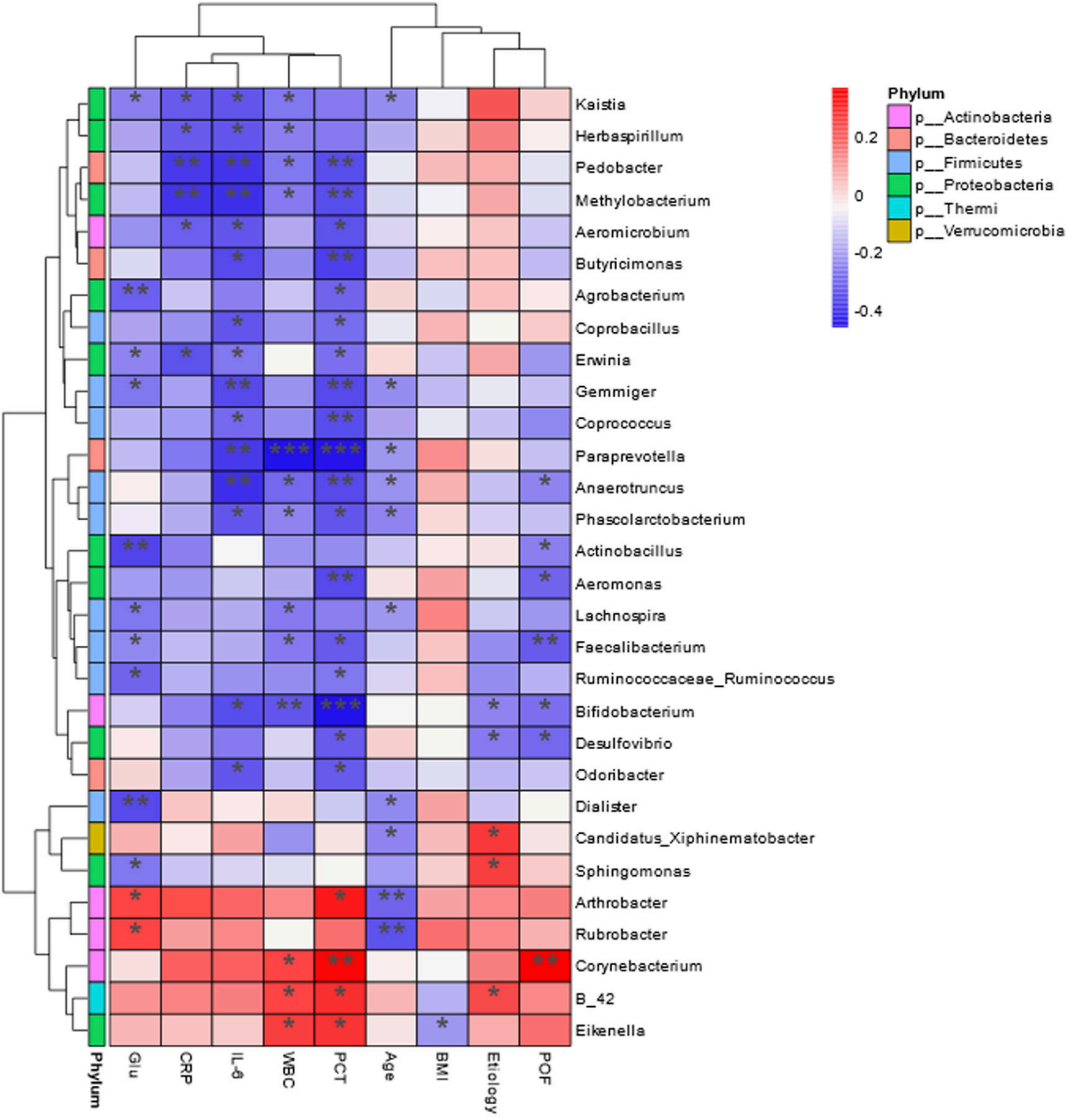
Heat map of Spearman’s correlation analysis. The environmental factors on the X-axis and species on the Y-axis. The R-values (rank correlation) and P-values were obtained by calculating the R-values, which are shown in different colors, and P-values less than 0.05 are marked with *. The right-hand legend shows the color intervals of the different R-values, while the color bar on the left side indicates the phylum to which the species belongs (*P < 0.05; **P < 0.01; ***P < 0.001).

Correlation heatmap analysis revealed significant associations between clinical parameters and bacterial genera (Figure 9). Age was significantly negatively correlated with *Arthrobacter* and *Rubrobacter* (*P* < 0.01). BMI showed a negative correlation with *Eikenella* (*P* < 0.05). White blood cell count was negatively correlated with *Bifidobacterium* (*P* < 0.01) and *Paraprevotella* (*P* < 0.001). Blood glucose levels were negatively correlated with *Dialister*, *Actinobacillus*, and *Agrobacterium* (*P* < 0.01). IL-6 levels were significantly negatively correlated with *Pedobacter*, *Methylobacterium*, *Gemmiger*, *Prevotella*, and *Anaerotruncus* (*P* < 0.01). CRP levels were negatively correlated with *Pedobacter* and *Methylobacterium* (*P* < 0.01). PCT levels were negatively correlated with *Corynebacterium*, *Bifidobacterium*, *Aeromonas*, *Paraprevotella*, and *Anaerotruncus* (*P* < 0.01). Persistent organ failure was positively correlated with *Corynebacterium* and negatively correlated with *Faecalibacterium* (*P* < 0.01).

### Functional variability analysis between groups

**FIGURE 10:**
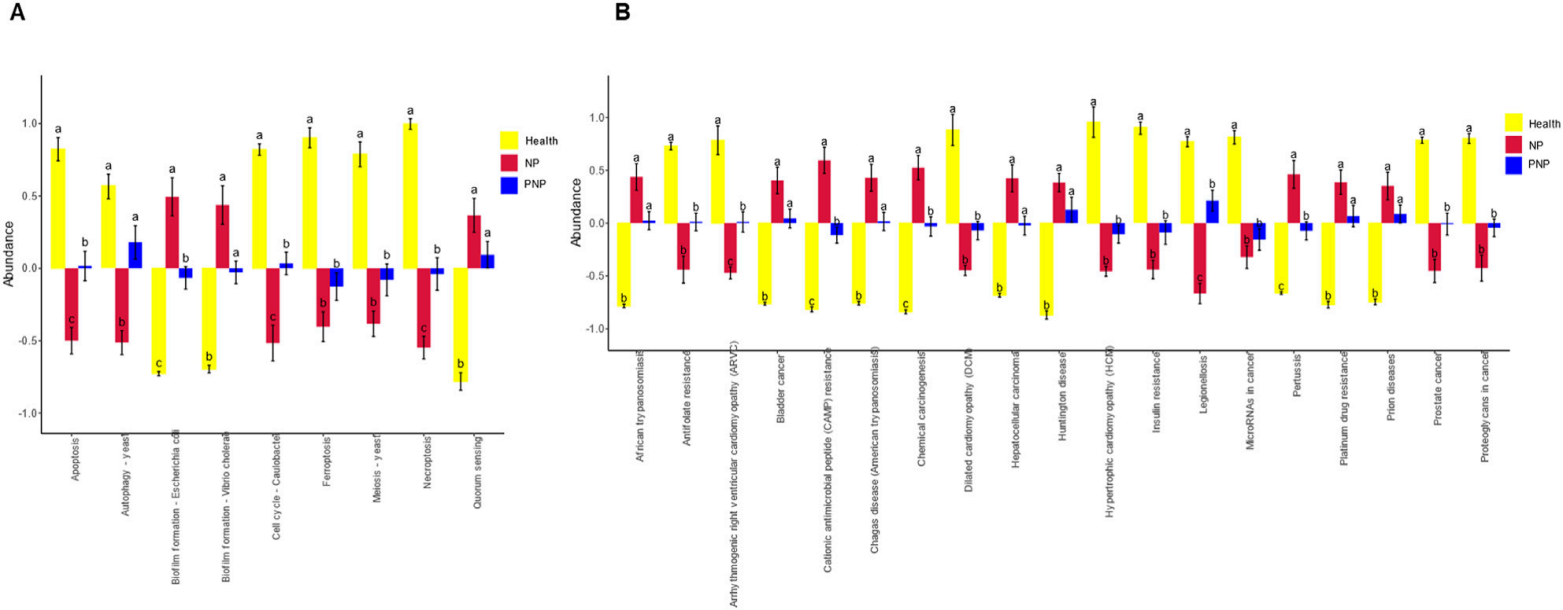
Analysis of functional differences in GM between groups **(A)** Cellular Processes pathways with all significant differences obtained by ANOVA and Duncan’s test; **(B)** Human Diseases pathways with all significant differences obtained by ANOVA and Duncan’s test; horizontal coordinates are pathway names; for each pathway, different colors are used to indicate For each pathway, different groupings are indicated by different colors, and if there are the same letters above the two groupings, it means the difference is not significant, otherwise the difference is significant.

After functional annotation, the information of grouping was combined to clarify the functional differences of GM between different groups. In predicting cellular processes, significant differences were observed in *Apoptosis*, *Biofilm formation - Escherichia coli*, *Cell cycle - Caulobacter*, and *Necroptosis*. In terms of predicting Human Diseases, Functional annotation revealed significant differences between groups in cellular processes. The groups exhibited significant differences in pathways associated with *Arrhythmogenic right ventricular cardiomyopathy (ARVC)*, *Cationic antimicrobial peptide (CAMP) resistance*, *Chemical carcinogenesis*, *Legionellosis*, *Pertussis*, and *Proteoglycans in cancer* (Figure 10).

## Discussion

In this study, we found that 29.42% of NP patients developed pancreatic endocrine insufficiency, while 19.12% exhibited PEI. Compared with healthy controls, the GM composition of NP and PNP patients showed significant differences. Specifically, NP patients exhibited a reduction in beneficial bacterial genera, such as *Roseburia*, *Ruminococcaceae*, and *Bacteroidaceae*, alongside an increase in opportunistic pathogenic genera, including *Bacillales*, *Streptococcus*, and *Enterobacter*. Similarly, in PNP patients, probiotic bacteria such as *Faecalibacterium prausnitzii* and *Bacteroides ovatus* displayed an upward trend in abundance.

Post-pancreatitis immune system activation can alter the microenvironment and intestinal permeability in the gut, leading to a reduction in the diversity of the intestinal flora. At the same time, PEI is often accompanied by a decrease in duodenal pH, changes in the intestinal flora, and subsequently affects glucose metabolism, inducing intestinal bacterial overgrowth and dysbiosis (Petrov, 2019; Guo et al., 2024). Research by others has confirmed that patients with pancreatic endocrine insufficiency have unique bacterial species characteristics compared to those with T1DM, T2DM, and healthy controls, including *Nesterenkonia* sp. *AN1, Clostridium magnum, Acinetobacter lwoffii, Clostridium septicum, Porphyromonas somerae, Terrabacter tumescens, and Synechococcus sp* (Talukdar et al., 2021). In this study, although the composition of the intestinal flora in the NP group and the PNP group was different, the incidence of exocrine and endocrine pancreatic insufficiency was similar in the long-term follow-up of both groups, indicating that *F. prausnitzii and Bacteroides ovatus* are not specific markers for assessing pancreatic exocrine and endocrine insufficiency. This might be related to the fact that we did not regularly collect fecal samples from patients during the follow-up process and did not conduct subgroup analyses based on whether NP patients had exocrine and endocrine insufficiency of the pancreas.

Previous studies (Zhu et al., 2019; Yu et al., 2020; Zhang et al., 2025) have reported dysbiosis in the intestinal flora of patients with acute pancreatitis (AP), with significant GM compositional changes correlating to disease severity (MAP, MSAP, SAP). However, most studies have been limited to single time-point analyses, lacking long-term follow-up data on GM dynamics. To address this gap, we performed 16S rRNA sequencing on stool samples from healthy controls, onset NP patients, and PNP patients. Our findings provide robust evidence regarding GM changes during NP recovery, which may inform strategies for preventing long-term complications and improving NP patient quality of life.

Alpha and beta diversity metrics were employed to assess GM composition and diversity across the study groups. Alpha diversity indices (Chao1, Shannon, and Simpson) revealed lower diversity and richness in the onset NP group compared to the recovery group (*P* < 0.05) and healthy controls (*P* < 0.001). Beta diversity analyses (PCA and PCoA) further confirmed significant compositional differences between groups. Reduced GM diversity during NP onset reflects the destabilization and poor resilience of the gut flora due to pancreatic inflammation, leading to a compromised intestinal microbiome (Fassarella et al., 2021). Stabilized GM, characterized by a diverse and balanced composition, enhances host resistance to pathogenic invasion and opportunistic bacterial overgrowth (Buffie and Pamer, 2013).

In line with prior research, AP-associated dysbiosis was characterized by disrupted intestinal barrier function, reduced probiotic abundance (*Bacteroides*, *Prevotella*, and *Rothia*), and increased pathogenic bacteria (*E. coli-Shigella*, *Bacteroides immobilis*, and *Clostridium difficile*) (Zhu et al., 2019). Among the common GM species, *F. prausnitzii* and *Akkermansia muciniphila* were prevalent in healthy controls, while *Bacteroides ovatus* and *Bacteroides uniformis* dominated in the NP group. Recovery in the PNP group was marked by higher abundances of *F. prausnitzii* and *B. ovatus*, approaching levels observed in healthy controls. This compositional shift may contribute to the gradual restoration of gut homeostasis. Notably, *F. prausnitzii* plays a key role in intestinal health by producing butyrate, which suppresses inflammation via NF-κB inhibition (Lopez-Siles et al., 2017). Similarly, *B. ovatus* generates short-chain fatty acids (SCFAs) such as acetate and propionate, which reduce inflammation by stimulating enteroendocrine cells and inhibiting histone deacetylases, SCFAs can enhance intestinal barrier function and participate in host immune regulation (Markowiak-Kopeć and Śliżewska, 2020; Horvath et al., 2022). Compared with mild and moderately severe AP patients, all species with differential abundance that were overexpressed in SAP patients belonged to the taxon of common producers of short-chain fatty acids (SCFA), and functional pathways that promote SCFA production were more expressed in rectal samples from SAP patients (Ammer-Herrmenau et al., 2024). These findings suggest that increased abundance of probiotics such as *F. prausnitzii* and *B. ovatus* in PNP patients may contribute to improved gut health.

Correlation analyses revealed significant negative relationships between inflammatory markers and specific probiotic genera. *Bifidobacterium* was negatively correlated with WBC count and serum PCT levels, while *Paraprevotella* was negatively associated with WBC count, IL-6, PCT levels, and persistent organ failure. These results align with previous studies linking AP-induced intestinal barrier disruption to reduced probiotic populations, translocation of endotoxins, and elevated inflammatory factors, which exacerbate complications such as SIRS, abdominal infections, and sepsis (Ammori et al., 2003; Tan et al., 2015; Zhu et al., 2019). Han et al. further demonstrated that *Bifidobacterium* and its metabolite lactate reduce inflammation in AP models via TLR4/MyD88- and NLRP3/caspase-1-dependent pathways (Li et al., 2022).

Functional analysis indicated that gut strains in the NP group were associated with pathways involving necroptosis, apoptosis, and various disease processes, such as arrhythmogenic right ventricular cardiomyopathy and cationic antimicrobial peptide resistance. Increased apoptosis in intestinal epithelial cells corresponded to a rise in opportunistic pathogens (*Salmonella*, *Shigella*, and *E. coli*), further aggravating intestinal barrier damage (Günther et al., 2013). In AP rats, necroptosis inhibitors, such as Nec-1, have shown potential in mitigating intestinal damage and barrier dysfunction (Cui et al., 2019), For Hypertriglyceridemic pancreatitis (HTGAP) mouse model, *Bacteroides uniformis* in the intestine and its metabolite taurine can accelerate the formation of neutrophil extracellular capture NETs (NETs) by activating the NF-κB and IL-17 signaling pathways, ultimately exacerbating pancreatic injury (Li et al., 2023), highlighting a promising avenue for future research.

The study has several limitations, for instance, it was conducted at a single center, with no external validation. While the incidence of PEI and pancreatic endocrine insufficiency was determined, the risk factors and specific microbiota for these complications remain unclear. Interventions, such as probiotic supplementation or pancreatic enzyme replacement, were not implemented, limiting insights into their effects on long-term outcomes. In addition, the 16S rRNA sequencing depth was insufficient for high-resolution species-level annotation, restricting functional and mechanistic analyses of the microbiota.

## Conclusion

This study revealed that 29.42% of NP patients developed pancreatic endocrine insufficiency, while 19.12% had PEI. The onset NP group exhibited distinct GM changes, dominated by *B. ovatus* and *B. uniformis*, whereas recovery in the PNP group was marked by increased *F. prausnitzii* and *B. ovatus* abundance. Probiotic genera such as *Bifidobacterium* and *Prevotella* showed diagnostic and predictive potential due to their negative correlations with inflammatory markers. These findings provide a foundation for exploring GM-based diagnostic, preventive, and therapeutic strategies for NP patients.

## Data Availability

The datasets presented in this study can be found in online repositories. The names of the repository/repositories and accession number(s) can be found below: https://www.ncbi.nlm.nih.gov/, PRJNA1117862.
