# Supplementary material for: Dynamic relationship between gut microbiota and post-necrotizing pancreatitis: insights from a multi-stage 16S rRNA sequencing study
Source: Front Pharmacol. 2025 May 22;16:1577558. doi: 10.3389/fphar.2025.1577558 (PMC12137087; doi:10.3389/fphar.2025.1577558)
Supplement: Supplementary file 1 [file Supplementaryfile1.docx]

**Supplementary material**

**Dynamic Relationship Between Gut Microbiota and Post-necrotizing Pancreatitis: Insights from a Multi-Stage 16S rRNA Sequencing Study**

Jiongdi Lu^1,2#^, Zhe Wang^1,2#^, Feng Cao^1,2^, Jia Li^1,2^, Guofeng Ji^1,2*^, and Fei Li^1,2*^

^1^Department of General Surgery, Xuanwu Hospital, Capital Medical University, Beijing, China

^2^Clinical Center for Acute Pancreatitis, Capital Medical University, Beijing, China

* **CORRESPONDENCE:**

Guofeng Ji, Email: jigf1227@163.com

Fei Li, Email: feili36@ccmu.edu.cn

**^#^** These authors contributed equally to this work and share first authorship.

| **Observation indicators** | **Definition** |
| --- | --- |
| Acute pancreatitis ^[13]^ | Fulfillment of two of the following three criteria: 1) acute onset of epigastric pain radiating to the lower back; 2) blood amylase and/or lipase levels >3 times higher than normal; and 3) imaging examination (e.g., abdominal ultrasound, enhanced CT, and MRI) revealing typical findings of acute pancreatitis. |
| Necrotizing pancreatitis ^[14]^ | Presence of varying density shadows in the pancreatic parenchyma on contrast-enhanced CT, with no enhancement in the pancreatic parenchyma in the early stages of disease. The degree of pancreatic necrosis in necrotizing pancreatitis patients was divided into <30 %, 30–50 %, and > 50 %. |
| Infected pancreatic necrosis ^[13]^ | Fulfillment of either of the following two criteria: 1) abdominal enhanced CT scan displaying the "bubble sign" in pancreatic and/or peripancreatic tissues; 2) development of positive pancreatic necrotic bacterial or fungal cultures with fine-needle aspiration (FNA) or other micro-invasive procedures. |
| Organ failure |  |
| Pulmonary failure | PaO_2_/ FIO_2_ <300, or need for mechanical ventilation. |
| Circulatory failure | Circulatory systolic blood pressure <90 mm Hg, despite adequate fluid resuscitation, or need for inotropic catecholamine support. |
| Renal failure | Creatinine level ≥177 μmol/L after rehydration or new need for hemofiltration or hemodialysis. |
| New-onset organ failure | First onset of organ failure requiring intervention at any time in a 24-hour period. |
| Multiple organ failure | Number of organs in failure ≥2. |
| Long-term complications |  |
| Incision hernia | After patient discharge, the full-thickness abdominal wall is discontinuous and abdominal contents bulge, with or without obstruction |
| Pancreatic pseudocyst ^[2]^ | Mature, encapsulated collection (s) of fluid with a well-defined wall outside the pancreas, homogenous fluid density, no solid component |
| Recurrent pancreatitis | A history of two or more episodes with and interval of at least 3 months |
| Pancreatic exocrine dysfunction | Clinical symptoms were improved by oral pancreatic enzyme use for more than 6 months, with no need to take this drug before the onset of AP |
| Pancreatic endocrine dysfunction | New onset diabetes after pancreatitis, need oral hypoglycemic drugs or insulin therapy for at least 6 months |
| Chronic pancreatitis ^[15]^ | Patients experience abdominal pain, weight loss, diabetes, and fatty diarrhea, endosonography/CT/MRI imaging shows dilated main duct and side branches, intraductal calcifications, parenchymal calcifications. The symptoms did not occur before the onset of AP |

**TABLE S1 Definitions of the** **observation indicators**

**TABLE S2: Characteristics of the cohort population**

| **Characteristics** | **Cohort population**  **（n=88）** |
| --- | --- |
| Gender [n(%)] |  |
| male | 50（50） |
| female | 38（50） |
| Age, years (M±SD) | 48.26±17.91 |
| BMI (Kg/m^2^） | 24.92±4.58 |
| Pre-existing comorbidities [n (%)] |  |
| Hypertension | 24（27.27） |
| Coronary heart disease | 7（7.95） |
| Diabetes | 14（15.91） |
| Others | 27（30.68） |
| Smoking [n(%)] | 13（14.77） |
| Drinking [n(%)] | 19（21.59） |

**TABLE S3: Comparison of clinical outcomes between two groups**

| **Characteristics** | **NP group**  **（n=34）** | **PNP group**  **（n=34）** | ***P*-value** |
| --- | --- | --- | --- |
| POF [n(%)] |  |  | 0.587 |
| Single OF | 10（29.41） | 12（35.29） |  |
| Multiple OF | 3（8.82） | 2（5.88） |  |
| Type of OF |  |  |  |
| Renal Failure | 6（17.64） | 5（14.71） | 0.883 |
| Respiratory failure | 3（8.82） | 5（14.71） | 0.458 |
| Circulatory Failure | 4（11.76） | 5（14.71） | 0.562 |
| IPNpatients [n(%)] | 23（67.65） | 20（58.82） | 0.316 |
| Nutritional support [n(%)] |  |  | 0.198 |
| Only parenteral nutrition | 16（47.06） | 11（32.35） |  |
| Combined nutrition | 18（52.94） | 23（67.64） |  |
| Duration of nutritional support [days (mean±SD)] | | | |
| Parenteral nutrition | 25.47±18.02 | 26.81±7.85 | 0.685 |
| Enteral nutrition | 34.48±25.84 | 38.76±8.09 | 0.736 |
| Surgical intervention [n (%)] | 17 （50.00） | 20（58.82） | 0.413 |
| Number of operations [time median(range)] | 2（1-4） | 2（1-5） | 0.224 |
| Surgical complications [n (%)] |  |  | 0.215 |
| Intraabdominal hemorrhage | 0（0） | 1（2.94） |  |
| Gastrointestinal fistula | 1（2.94） | 0（0） |  |
| Gastrointestinal obstruction | 0（0） | 2（5.88） |  |
| Others | 1（2.94） | 2（5.88） |  |
| ICU stay [days (mean±SD)] | 33.39±19.25 | 35.75±25.38 | 0.771 |
| Hospital stay [days (mean±SD)] | 42.62±37.56 | 54.71±41.37 | 0.189 |

**TABLE S4: Quality of life rating scale during the follow-up period of patients**

| **Rating scale (Mean ± SD)** | **NP group（n=34）** | **PNP group（n=34）** | ***P*-value** |
| --- | --- | --- | --- |
| SF-36 Physical health score^1^ | 36.04±16.69 | 37.89±11.34 | 0.64 |
| SF-36 Mental health score^1^ | 37.56±19.67 | 41.89±12.56 | 0.345 |
| EQ-5D based health status score^2^ | 61.56±28.38 | 67.96±20.66 | 0.354 |
| Lzbicki pain score^3^ | 17.68±14.83 | 18.89±13.03 | 0.756 |

**TABLE S5: Abundance of major strains in the three groups**

| **Phylum level** | **Health group** | **NP group** | **PNP group** | ***P*-value** |
| --- | --- | --- | --- | --- |
| Firmicutes | 0.449±0.113 | 0.433±0.239 | 0.469±0.187 | 0.508 |
| Bacteroidetes | 0.428±0.128 | 0.258±0.194 | 0.304±0.158 | 0.001^*^ |
| Proteobacteria | 0.059±0.026 | 0.252±0.228 | 0.164±0.133 | 0.002^*^ |
| Actinobacteria | 0.027±0.012 | 0.020±0.0231 | 0.029±0.027 | 0.06 |
| Verrucomicrobia | 0.007±0.015 | 0.023±0.070 | 0.030±0.095 | 0.492 |

**TABLE S6: Abundance of major strains in the three groups**

| **Class level** | **Health group** | **NP group** | **PNP group** | ***P*-value** |
| --- | --- | --- | --- | --- |
| Bacteroidia | 0.423±0.133 | 0.256±0.197 | 0.295±0.162 | 0.002^*^ |
| Clostridia | 0.422±0.109 | 0.212±0.135 | 0.331±0.173 | 0.001^*^ |
| Gammaproteobacteria | 0.022±0.015 | 0.241±0.233 | 0.138±0.126 | 0.001^*^ |
| Bacilli | 0.019±0.018 | 0.206±0.293 | 0.133±0.249 | 0.001^*^ |
| Verrucomicrobiae | 0.006±0.016 | 0.023±0.070 | 0.030±0.096 | 0.012^*^ |

**TABLE S7: Abundance of major strains in the three groups**

| **Order level** | **Health group** | **NP group** | **PNP group** | ***P*-value** |
| --- | --- | --- | --- | --- |
| Bacteroidales | 42.34±13.27 | 25.64±19.77 | 30.93±18.11 | 0.002^*^ |
| Clostridiales | 42.27±10.97 | 21.24±13.59 | 30.59±16.70 | 0.001^*^ |
| Enterobacteriales | 1.32±1.10 | 22.97±23.70 | 13.77±18.49 | 0.001^*^ |
| Lactobacillales | 1.79±1.81 | 19.70±29.29 | 12.11±23.74 | 0.001^*^ |
| Verrucomicrobiales | 0.621±1.65 | 2.33±7.00 | 2.22±7.45 | 0.12 |

**TABLE S8: Abundance of major strains in the three groups**

| **Family level** | **Health group** | **NP group** | **PNP group** | ***P*-value** |
| --- | --- | --- | --- | --- |
| Bacteroidaceae | 29.286±9.738 | 17.057±13.408 | 20.838±13.685 | 0.004^*^ |
| Enterobacteriaceae | 1.328±1.097 | 22.978±23.703 | 13.772±18.493 | 0.001^*^ |
| Lachnospiraceae | 17.053±6.754 | 9.728±8.170 | 13.235±8.934 | 0.003^*^ |
| Ruminococcaceae | 17.830±6.509 | 6.864±5.839 | 11.012±8.241 | 0.001^*^ |
| Enterococcaceae | 0.828±1.026 | 16.338±27.519 | 9.818±22.764 | 0.001^*^ |

**TABLE S9: Abundance of major strains in the three groups of patients**

| **Genus level** | **Health group** | **NP group** | **PNP group** | ***P*-value** |
| --- | --- | --- | --- | --- |
| Bacteroides | 29.281±9.730 | 17.057±13.408 | 19.651±14.138 | 0.004^*^ |
| Parabacteroides | 3.407±2.046 | 2.232±2.601 | 3.063±4.085 | 0.016^*^ |
| Enterococcus | 0.826±1.023 | 16.335±27.518 | 8.558±22.670 | 0.001^*^ |
| Faecalibacterium | 10.558±5.711 | 3.141±3.283 | 6.199±6.029 | 0.001^*^ |
| Prevotellaceae_Prevotella | 5.257±10.773 | 2.373±4.823 | 2.486±5.911 | 0.317 |

**TABLE S10: Abundance of major strains in the three groups of patients**

| **Species level** | **Health group** | **NP group** | **PNP group** | ***P*-value** |
| --- | --- | --- | --- | --- |
| prausnitzii | 10.549±5.713 | 3.137±3.279 | 6.194±6.027 | 0.001^*^ |
| uniformis | 7.037±5.694 | 2.178±2.392 | 2.642±2.391 | 0.001^*^ |
| ovatus | 4.675±3.390 | 2.182±1.998 | 3.104±2.591 | 0.017^*^ |
| muciniphila | 0.613±1.643 | 2.328±7.001 | 3.014±9.677 | 0.094 |
| copri | 4.995±10.440 | 0.552±1.327 | 1.639±5.856 | 0.001^*^ |
